# Digestion of Albuminous Matters by the Pancreas

**Published:** 1859-10

**Authors:** Lucien Corvisart


### ECLECTIC DEPARTMENT,

### AND SPIRIT OF THE MEDICAL PERIODICAL PRESS.

---

*Digestion of Albuminous Matters by the Pancreas.* By Lucien Corvisart.

Most of our readers will remember our recent notice of the paper of M. Corvisart. His essay has attracted much attention and some hostile criticisms. The following remarks by him upon his reviewers and experimental, or other critics, explain some parts of his essay which were not altogether clear.

As regards certain experiments by Kefestein and Halwach, from which these gentlemen obtained results which they looked upon as adverse to the views of M. Corvisart, he speaks thus :—

The work presented by these gentlemen to the Academy of Sciences at Göttingen terminates with this conclusion : We entirely dissent from the views of M. Corvisart. The pancreatic juice is incapable of dissolving albumen. Now, to the dictum of these gentlemen it would have been easy to oppose the results of my numerous experiments, which have been subjected to the strictest verification. Nevertheless, I determined upon offering to the Academy my attacked essay, and solicited them to accept for my reply an account of one experiment only, the details of which are as follows :—

The subject of the experiment was a young dog weighing about "twenty-four pounds," and previously deprived for the space of fifteen hours of both solid and liquid food : 34 grammes of hardened white of eggs, boiled in water for fifteen minutes, separated from the shells and yolk and roughly crumbled in a cloth, were put into the duodenum, and ligatures placed around its first and third portions. For the purpose of bringing about digestion simultaneously in the stomach, 20 grammes of the same albumen were placed in that viscus, all egress to the surface being prevented by the ligature at the first portion of the duodenum, and by another ligature placed around the clavical termination of the œsophagus. During the operation the pancreas was not disturbed in the slightest degree ; indeed it was not even seen. Tubes were employed for the purpose of introducing the albumen at the same instant into the stomach and intestine ; and all these operative precautions to which reference is made at page 9 of my essay, and which seemed to me indispensable to the success of the operation, were most scrupulously observed. Fifteen hours afterwards, the dog was killed by strangulation. The duodenum presented a swollen, red, and turgid appearance ; taken out of the abdominal cavity, and emptied of its contents, we found 150 grammes of a viscous liquid, of neutral, or at most of a feebly-alkaline reaction, and altogether destitute of putrefactive odor. The intestine displayed no traces of the coagulated albumen placed within it in the first instance, if we except five or six soft and attenuated fragments, recognizable, it is true, but not amounting in weight to so much even as four grammes.

(*a*) It follows, therefore, that the mixed fluid in the duodenum is capable of digesting albumen.

The stomach, in the above experiment, was found to contain 250 grammes of an acid liquid. The hard albumen had, in like manner, disappeared by solution.

The pancreas of the same dog, examined when the digestive process was at its acme, both in the stomach and in the duodenum, presented the following features: The color was of a faint pink. It showed no signs of tearing, or of ecchymosis. It was removed, cut up into small pieces, and placed in 200 grammes of water, maintained for twenty-four hours in a closed glass vessel, at a temperature varying from 7° to 12° Cent.; the product was then filtered, and I collected 180 grammes of a reddish, viscous fluid, which displayed neither a marked acidity nor alkalinity, to test-paper of extreme sensibility. This pancreatic infusion was tried upon ovalbumen, cooked as in the preceding experiment, and pounded. After remaining four hours only in the stove, at a temperature of 40° Cent., the number of grammes of albumen which had disappeared amounting to 45 of the albumen employed, in the first instance.

(*b*) Hence, it follows that a simple infusion of pancreas can, by a power of its own, and without any assistance from intestinal secretions, or bile, etc., digest a large quantity of coagulated albumen.

By the action of a few grammes of the infusion, I was enabled to assign to it a digestive power over fresh, uncooked fibrin, which, calculated proportionately for an infusion of the whole of a pancreas, would suffice for the digestion of 60 grammes of fibrin. Both the digestions, by means of pancreatic infusions and the vivisection itself, were performed in the presence of gentlemen at that time in Paris, viz.: Dr. Kuhne, pupil of Messieurs Wohler and Wagner, and Dr. Snellen, of Utrecht, pupil of M. Donders. (Extract from my letter to the Acad. des Sciences de Gött. Nachr., No. 6, March, 1859, and Zeitschrift für Rationellen Med. of Henle and Pfeuffer, 1859.) It will be remarked that these several quantities—45 grains of albumen, 60 of fibrin—amount as nearly as possible to within a few grammes of the quantities I had specified in my essay two years before. Messrs. Kefestein and Halwachs have nevertheless affirmed that their experiments were more exact than any others that had been performed. But the exactness of their proceedings began only at that stage when the stove came into requisition; while in fact it was of the highest importance that such exactness should begin in the very abdomen of the animal whose function they sought to determine. These gentlemen were not sufficiently careful in guarding against fallacy. Firstly, inasmuch as they employed the pancreatic juice of an animal unfortunately laboring for eight days under a fistula; secondly, as they made infusions of the pancreas without being mindful of selecting the gland at a fixed and suitable period of the digestive process.

First. I had forewarned experimentalists in my paper that the results obtained by means of the tubes appended to the excretory canal—that is to say, the pancreatic fistulæ—would be so various that it would be impossible to arrive at any definite conclusions by means of them. Messrs.

Kefestein and Halwachs continued to employ this mode of procedure in carrying out their first series of experiments. The result has been that they have secured none but negative results. They moreover placed themselves in conditions most unfavorable to success, by an ill-judged excess of experimental zeal, by which they assigned the preference both in the collecting the juice and in the carrying out the experiment to that secretion of the pancreas poured out subsequently to an irritation lasting eight days without intermission, and conveyed by the tube, over that secretion formed immediately after the performance of the operation.

It is self-evident that the pancreatic juice collected almost at the moment of the operation, alone approximates to the normal secretion, the first quantity which drains away being "that which was already secreted in its physiological integrity in the gland prior to the operation." The longer the interval permitted to elapse, the further does the secretion depart from the true physiological type. Every organ, in fact, possesses its own special sensibility. The eye cannot, like the mouth, tolerate the presence of a grain of sand. The pancreas in no way habituates itself to the existence of fistulæ, as does the stomach, designed, as is this latter, for the contact of foreign bodies.

On the one hand, pancreatic fistulæ, far from being capable of enduring for years, like those of the stomach, end fatally in a few days, or at most in a few weeks. On the other hand, in the case of a pancreatic fistula, the properties of the pancreatic juice begin to deteriorate considerably after a lapse of two, or at most of three days. This is owing either to a diminution in the weight of the solid constituents, or to an alteration in the properties of the secreted ferments without diminution of weight. By the eighth day the deterioration is at its maximum. At this time the pancreatic secretion is in the condition it assumes when it has been made to boil. It has lost all potency over albuminous bodies, although it is still capable of forming an emulsion with fat, and of imparting an alkaline reaction.

The mode of procedure adopted by Messrs. Kefestein and Halwachs with fistulæ, will always be productive of negative results.

It is indispensable, when we desire to obtain pancreatic juice as nearly as possible in its normal condition, that that secretion be chosen which was formed in the gland prior to vivisection—that is to say, "that" juice which flows forthwith upon the operation.

"Upon the fulfillment of this condition depends the superiority of the infusion of a pancreas" taken from an animal at the moment of killing it; for if the pancreas be removed within a few seconds after the animal has been killed, the infusion takes hold of the juice normally secreted during life, and which has not yet escaped from the gland.

Secondly. This proceeding has furnished the material for the second series of experiments of Messrs. Kefestein and Halwachs.

But here again they have erred in a most marked degree. It is not sufficient that a secreting organ be taken in order to obtain its secretion forthwith upon the death of the animal. The gland must be secured at that moment when its secreting activity is at its height. This precaution has been neglected by Messrs. Kefestein and Halwachs; and to this omission is to be ascribed the confirmation of the negative results they had obtained. With regard to the experiments detailed in my former paper, I may state that they were performed with infusions of the pancreas taken from animals in whom the duodenum and stomach were full at the moment of killing. M. Meissner has distinctly affirmed that he has obtained active infusions by taking the precaution of securing the pancreas during the period of digestion—this precaution is indispensable. I may add, that if a young and healthy dog be fed with mixed and abundant diet, and that if it be killed towards the fifth or six hour after the meal, and the pancreas be then removed, the infusion of the gland will possess the highest* digestive activity.†

There is in reality a fasting condition of the duodenum, which is not identical with the similar condition of the stomach; in like manner, the fasting condition of the stomach is not identical with that of the mouth. It is highly probable that some little fluid may escape from the pancreas immediately upon the reception of food from the stomach ; but the period of greatest glandular activity, of the highest efficiency of the pancreatic juice, is at that moment when the stomach, having performed its function, the duodenum begins to act in its turn. This period in the dog is attained toward the fifth or sixth hour. At that time, the stomach still contains some food, and the duodenum contains some already. Before this period is attained, the duodenum is still in its fasting condition, and the pancreas inactive ; subsequently to it again, the pancreas becomes exhausted.

Montègre went so far as ostensibly to deny the digestive action of the gastric juice, and even its acidity ; for the reason that he was accustomed to examine the secretion during the condition of fasting. The same error has induced Messrs. Kefestein and Halwachs to deny to the pancreatic juice all digestive power over albumen. Their good faith, it is to be observed, is entirely foreign to the question ;‡ whoever imitates them will see, like them, negative results.

Thirdly. Researches of M. Meissner upon the functions of the pancreas.

Following upon Messrs. Kefestein and Halwachs, Professor Meissner published in the "Zeitschrift für Rationellen Med." of April, 1859 (having previously read an account of them as early as the autumn of 1856, at the Scientific Congress of Carlsruhe,) the experiments which led him to affirm most positively not only the solution of albuminous bodies by the pancreas,

---

* At such a period of digestion, the pancreatic juice is so energetic, that if one omits to arrest the infusion of this gland at the proper time, this latter, if it have been cut up in very small pieces, in part disappears, dissolved and digested by its own proper juice which has escaped from the channels in which normally it is confined during life.

† The infusion made in compliance with these conditions, is frequently able to digest 20 or 30 grammes of fibrin in the cold, and in but a few hours (10° Cent.).

‡ I would add, that on preparing for purposes of study an infusion of the pancreas, it is necessary to avoid crushing the gland, or agitating it too frequently in the water, or protracting the infusion beyond the period when the liquor becomes clouded. Under all the circumstances, one perceives by this last sign that the juice is beginning to act upon the fatty matters in the gland itself; at a later period it will have begun to act upon the azotized substance, since, like the gastric juice, the pancreatic juice exhausts itself by agitation. Commonly, an infusion which is long in filtering and is cloudy, has partly lost its efficiency, unless the operation is carried on at a very low temperature—seven to eight degrees Cent. Rapidity is the rule in the preparation of the infusion.

apart from all putrefaction, but the transformation of these bodies into peptone, as I had previously maintained. M. Meissner states: The results at which I have arrived completely confirm those obtained by M. Corvisart, with this restriction, that it is necessary that the pancreatic juice be acid, and not neutral, alkaline, or acid indifferently.

I had in fact stated in the ninth proposition,—"The pancreatic juice possesses the special property of acting efficiently, whether in the alkaline, neutral, or acid state."

I beg to refer to pages 8, 29, 32, and 33, of my essay, where is detailed the digestion of albumen brought about either naturally in the duodenum, or by employing the stove with pancreatic juice by itself, and most effectively performed, the reaction being neutral and even alkaline; and I would add, that I was led to assert the existence of this neutrality, not only from my belief in its verification, as far as albumen was concerned, but inasmuch as my experiments on digestion, repeated on fibrin, (pp. 36, 40, 42,) cellular tissue and gelatine, (pp. 67 and 78,) on muscular tissue and on caseine, (pp. 92, 98,) were attended by similar results. Moreover, in these comparative experiments I was not dealing with imponderable quantities, quantities difficult to estimate, but with 20, 30, or 40 grammes of the articles of food, the digestion of which was completed under the influence of an infusion of a dog's pancreas, alkaline, acid, or neutral. The object of M. Meissner led me to investigate anew whether the words "equally well" of my ninth proposition were rigorously correct. I have consulted the records of my experiments. I have taken into consideration the figures indicating the weight of albumen digested by some of the same pancreatic juice, (but varied in such a manner that one solution was neutral, a second alkaline, a third acid.) I noticed differences, it is true, but these amounted at most to a few grammes, and were so slight in themselves, that at this moment it would be impossible for me to say whether, forty grammes of albumen being experimented upon, four grammes more are digested by the pancreatic secretion being acid or alkaline. This indifference was likewise displayed on placing food in the closed duodenum. At the moment the animal was killed, the reaction was found to be at one time acid, at another neutral, and a third time alkaline; the weight of the material digested under those varying conditions being subject to but little alteration. In conclusion, I would remark that during the experiment, the minutes of which have been drawn up, the attention of Messrs. Kuhne, Snellen, and myself was specially directed to the point in dispute. The minutes run as follows:—

With regard to the duodenum.—"The duodenum was found to contain 150 grammes of a viscous liquid, neutral or but very faintly alkaline, having no putrefactive smell, and showing no traces of the 34 grammes of the coagulated albumen placed within it in the first instance, with the exception of five or six soft fragments, which, though recognizable, did not amount in weight to as much as 4 grammes." With regard to the pancreatic infusion.—"After remaining four hours in the stove, the quantity of solid albumen which had disappeared amounting to 45 grammes of the albumen originally employed;" and further, it is stated that "before the albumen was used the infusion displayed, to litmus or turmeric papers of great delicacy, no noticeable traces either of acidity or alkalinity."

The weight attaching to the researches of M. Meissner urges me to solicit most earnestly for further investigations, which doubtless will not fail to be productive of some explanation of the cause of the discrepancy to which this special point has given rise between us.—(*Lancet*, No. 25, vol. i., 1859.)

